# The Toxicological Impact of the Ultraviolet Filter Oxybenzone on Antioxidant Profiles in In Vitro Cultures of *Lentinula edodes*

**DOI:** 10.3390/toxics13030145

**Published:** 2025-02-20

**Authors:** Agata Kryczyk-Poprawa, Adrián Sánchez-Hidalgo, Wojciech Baran, Ewa Adamek, Katarzyna Sułkowska-Ziaja, Katarzyna Kała, Bożena Muszyńska, Włodzimierz Opoka

**Affiliations:** 1 Department of Inorganic and Pharmaceutical Analytics, Faculty of Pharmacy, Jagiellonian University Medical College, 30-688 Kraków, Poland; adri.91.3c@gmail.com (A.S.-H.); wlodzimierz.opoka@uj.edu.pl (W.O.); 2 Department of General and Analytical Chemistry, Faculty of Pharmaceutical Sciences in Sosnowiec, Medical University of Silesia in Katowice, Jagiellońska 4, 41-200 Sosnowiec, Poland; wbaran@sum.edu.pl (W.B.); eadamek@sum.edu.pl (E.A.); 3 Department of Medicinal Plant and Mushroom Biotechnology, Faculty of Pharmacy, Jagiellonian University Medical College, 30-688 Kraków, Poland; katarzyna.sulkowska-ziaja@uj.edu.pl (K.S.-Z.); k.kala@uj.edu.pl (K.K.); bozena.muszynska@uj.edu.pl (B.M.)

**Keywords:** mycoremediation, UV filter toxicity, oxybenzone, antioxidants, *Lentinula edodes*, biodegradation products

## Abstract

A detailed understanding of the toxic effects of organic UV filters, such as oxybenzone, on living organisms is crucial for assessing the feasibility of bioremediation methods. Due to the widespread use of oxybenzone as an ultraviolet filter in sunscreens, it has become an emerging contaminant of concern in the environment. This concern extends to fungi, which have the potential to neutralize a wide variety of xenobiotics released into the environment. The primary objective of the study was to elucidate the alterations of antioxidant profiles of the white-rot fungus *Lentinula edodes* in response to oxybenzone exposure. Samples with oxybenzone at a final concentration of 0.1 mg mL^−1^ were cultured in vitro with the mycelium of *L. edodes* for 14 days. The contents of the following antioxidant compounds were assessed: indole derivatives (6-methyl-D,L-tryptophan, tryptophan), ergothioneine, and phenolic acid (p-hydroxybenzoic acid), as well as lovastatin and ergosterol. The addition of oxybenzone negatively affected biomass growth, reducing it from 3.205 ± 0.4022 g to 0.5803 ± 0.1019 g. A considerable reduction in oxybenzone amounts was found in the medium after incubation (from 25 mg to 0.2993 ± 0.1934 mg). After lyophilization, the mycelium contained 1.1591 ± 0.0323 mg of oxybenzone. Additionally, eleven biotransformation products were assessed in the mycelium and medium samples using UPLC-Q ToF. After incubation, the transformation products were identified based on monoisotopic molecular mass and fragmentation spectra. The observed increase in the content of some antioxidants, e.g., ergothioneine, while reducing the content of others, such as lovastatin, suggests that the impact of xenobiotics on the antioxidant profile of in vitro cultures of *L. edodes* is complex. Marked alterations in biomass growth suggest a potential toxicological risk associated with oxybenzone. This study contributes to the understanding of the environmental impact of UV filters and emphasizes the need for safer alternatives.

## 1. Introduction

The dangers associated with UV radiation exposure have increased the use of cosmetic products containing UV filters. Sun protection products, which absorb or block UV radiation, are essential for preventing sunburn and reducing the risk of skin cancer. One of the most common organic UV filters is oxybenzone (2-hydroxy-4-methoxybenzophenone, benzophenone-3, BP-3) [1,2,3]. This compound was first synthesized in Germany in 1906 by chemists B. König and Stanisław Kostanecki. It is an old sunscreen ingredient developed by the now-defunct company General Aniline and Film Corporation (Wayne, NJ, USA) in the 1950s. Currently, it is sold under various trade names such as Milestab 9, Eusolex 4360, and Escalol 567, and it is used at concentrations of up to 6% in sunscreen preparations. It comes in the form of a pale yellow powder with a sweet odor. It belongs to the class of aromatic ketones known as benzophenones [4,5]. The most important property of oxybenzone is its ability to absorb UVA (320 to 340 nm) and UVB (290 to 320 nm) irradiation, which are mainly responsible for skin aging, sunburn, and the development of skin cancer. As a photoprotective agent, it has an absorption profile ranging from 270 to 350 nm, with two absorption peaks at 288 and 350 nm [6,7]. Similarly, UV filters are included as an ingredient in non-food plastic packaging and consumer products such as textiles and sports equipment to avoid much of the sun damage and discoloration of the products [8].

Despite its excellent photoprotective qualities, there is significant debate surrounding oxybenzone. Concerns have been raised about its potential side effects, including hormonal disruptions and photoallergic reactions. Some studies suggest that oxybenzone can act as an endocrine disruptor, potentially affecting hormone levels in the body [1,9,10]. These concerns have led to increased scrutiny and calls for more research to fully understand the safety and long-term effects of oxybenzone [11]. In 2008, scientists from the Centers for Disease Control and Prevention (CDC) showed the presence of oxybenzone in almost all urine samples tested (97% of 2517 participants). Generally, women and girls tend to have higher oxybenzone levels in their bodies compared to men and boys, likely due to the differing usage patterns of body care products like sunscreens [12].

The widespread use of oxybenzone as an ultraviolet filter in sunscreens has led to its emergence as an environmental contaminant of concern. According to Kim and Choi, oxybenzone has been identified in various environments, including water, soil, sediments, sludge, and living organisms [6]. The levels of UV filters in surface water exhibited significant seasonal fluctuations, with the highest concentrations observed during the summer months, likely due to increased recreational activities. Furthermore, it can react with chlorine to form hazardous by-products in pools and wastewater [13]. Oxybenzone exposure in plants has been linked to decreased photosynthesis, inhibited seed germination, and impaired growth [14,15]. Studies have shown that UV filters such as oxybenzone are stable against biodegradation or absorption, due to their lipophilicity. This could cause the accumulation of chemical compounds in the food chain that can result in an imbalance of ecosystems [16]. Environmentally, it harms coral, causing reef bleaching and mortality. C. Downs et al. found that oxybenzone can begin to damage coral at concentrations as low as 0.062 µg/L [17]. Numerous investigations have been carried out with the intention of verifying if it is a direct factor; this has led different governments, such as Hawaii’s, to ban oxybenzone, considering it as one of the factors causing bleaching of coral reefs [18].

Mycoremediation is a term coined by one of the most important mycologists today, Paul Stamets, in 1998. It is a form of bioremediation that takes advantage of fungi to degrade or retain pollutants in the environment. Mycelium is able to reduce xenobiotics from the environment. Some fungi have the ability to absorb and concentrate heavy metals. One of the main functions of fungi in the ecosystem is decomposition, which is carried out by the mycelium that secretes enzymes and extracellular acids capable of breaking down lignin and cellulose. These are organic compounds ‘similar’ to many organic pollutants. A large number of organic molecules are sensitive to the actions of various strains of white-rot fungi, including substances that are very persistent, such as polycyclic aromatic hydrocarbons. The key to mycoremediation is to identify which specific types of fungi are useful for removing (biodegradation, biosorption, and bioconversion) specific contaminants based on the environment, conditions, and viability [19,20,21,22,23].

*L. edodes* was selected for the study due to its proven efficacy in mycoremediation of xenobiotics such as cephalosporin antibiotics, synthetic testosterone, 17α-ethinyl estradiol, and azole antifungal agents [24,25]. *L. edodes* is an edible mushroom with a brown color and intense aroma, native to East Asia. The history of the classification of the fungus L. edodes, commonly called “shitake”, dates back to 1878. *L. edodes* produces many enzymes, such as peroxidase, cellulase, pectinase, xylanase, ligninase, and oxidase. Its extracellular enzymes could participate in the xenobiotic degradation process. Additionally, *L. edodes* mycelia have the ability to remove heavy metal contaminants through their accumulation and detoxification [26,27]. Rapid growth, the production of large amounts of biomass, and the wide distribution of hyphae in the environment promote the high efficiency of this fungi in the environmental cleaning process. The available literature lacks information on the bioremediation of oxybenzone and, more specifically for our study, bioremediation with fungi (mycoremediation). Therefore, the main objective of the present study was to evaluate the effectiveness of the use of the edible fungus *Lentinula edodes* (Berk) in the remediation of oxybenzone. The medium used for culturing *L. edodes* was enriched with oxybenzone to a final concentration of 0.1 mg mL^−1^. The levels of antioxidants such as indole derivatives (6-methyl-D,L-tryptophan, tryptophan), ergothioneine, and phenolic acid (p-hydroxybenzoic acid), as well as lovastatin and ergosterol, were assessed. Following the incubation period, the resulting biotransformation products were identified, and their potential toxicity risks were evaluated.

## 2. Materials and Methods

### 2.1. Reagents

Oxybenzone (pharmaceutical secondary standard) was obtained from Sigma-Aldrich Corp. (St. Louis, MO, USA). Merck (Darmstadt, Germany) supplied the HPLC-grade methanol, acetonitrile, 98% formic acid, chloramphenicol, and dichloromethane. Chemicals like glucose, maltose extract, casein hydrolysate, l-asparagine, adenine, and yeast extract were purchased from Sigma-Aldrich (St. Louis, MO, USA). Ammonium chloride, potassium dihydrogen phosphate, magnesium sulfate heptahydrate, calcium dichloride hexahydrate, iron trichloride, manganese sulfate monohydrate, and zinc sulfate hexahydrate were obtained from PPH Golpharm (Kraków, Poland). Water (quadruple-distilled) with a conductivity of less than 1 μS cm^−1^ was obtained using an S2-97A2 distillation apparatus (ChemLand, Stargard Szczeciński, Poland). All chemicals were of analytical grade.

### 2.2. Mushroom Material

For the study, the fruiting bodies of shiitake mushroom (*L. edodes* (BERK) Pegler mushroom) were obtained commercially from a local market in Poland (2019). Mycelial cultures were analyzed and registered in GenBank. The MN907099.1 GenBank accession number was granted to the analyzed nucleotide sequence of the *L. edodes* strain (https://www.ncbi.nlm.nih.gov/nuccore/MN907099.1, accessed on 1 January 2020). The Department of Medicinal Plant and Mushroom Biotechnology at Jagiellonian University Medical College in Kraków, Poland, houses the representative samples of the material used for further studies.

### 2.3. Preparation of L. edodes Mycelial Cultures

The mycelial culture was initiated using the fruiting bodies of *L. edodes*, and the resulting mycelium was used as the material for subsequent research. To prepare the mycelial culture of *L. edodes*, fragments of the hymenial part of the fruiting bodies were selected. Initially, these fragments were mixed with sterile redistilled water and transferred to BD Sabouraud Agar with chloramphenicol (antibacterial) under laminar airflow in a sterile room. The cultures were then incubated in a thermostat (ST500/B/40 Pol-Eko-Aparatura) at 23 °C ± 2 °C for two weeks. Mycelia obtained from the solid medium were used to establish initial cultures, which were then grown in liquid Oddoux medium (Oddoux, 1957). These cultures were shaken at 140 rpm on a rotary shaker (ALTEL, Lublin, Poland) and incubated at 23 °C ± 2 °C with a 12 h light (900 lx)/12 h dark cycle. The *L. edodes* cultures were maintained for two weeks before being subcultured. The *L. edodes* mycelium obtained was analyzed using the PCR method. The nucleotide sequence identified in this study was assigned GenBank accession number SUB6791903 Seq 1 MN907099.

### 2.4. Experimental Mycelial Cultures of L. edodes

In mycoremediation experiments, 25 mg of oxybenzone powder was introduced into 250 mL Erlenmeyer flasks containing sterile liquid medium (sterilization at 121 °C for 20 min) inoculated with *L. edodes* mycelial culture. Control samples, devoid of oxybenzone, were also prepared. Post-inoculation, the cultures were incubated in the shaker at a temperature of 23 °C ± 2 °C for 14 days. Subsequently, after two weeks of growth, the *L. edodes* biomass was isolated from the medium, washed with redistilled water, and freeze-dried using a lyophilizer (FreeZone 4.5, Labconco Corporation, Kansas City, MO, USA). The medium from each culture was evaporated to dryness using a vacuum rotary evaporator at 22 °C ± 2 °C. The freeze-dried biomass of each culture was weighed and ground in a mortar.

### 2.5. Sample Preparation for Analysis

Biomass (5 g of powdered mushroom material) was extracted using 125 mL of methanol in an ultrasonic bath at 49 kHz for 20 min (Sonic-2, Polsonic), and subsequently, the solution was filtered using a paper filter and a funnel and the methanol with the extract dripped into a flat glass container. The above steps were repeated three times. Approximately 375 mL of extract solution from each biomass was evaporated to dryness using a rotary vacuum evaporator at 22 °C ± 2 °C. Once dried, both the medium and biomass extracts were dissolved in 5–8 mL of methanol and filtered using 0.22 µm filters. All samples were equalized to 8 mL by adding methanol. The medium samples were analyzed directly, while the biomass samples, due to oxybenzone’s high concentration, were diluted in a 1:10 ratio with methanol and were analyzed via RP-HPLC analysis and UPLC.

### 2.6. HPLC Analysis

The analysis of organic compound content was performed chromatographically using the RP-HPLC-DAD method. This procedure relied on standard calibration curves, presuming a linear correlation between the peak area and the concentration of the reference standards.

The concentrations of indole compounds, phenolic compounds, lovastatin, ergothioneine, and *L*-phenylalanine were determined using RP-HPLC [28,29]. The concentration of these compounds was analyzed using a UV detector set at λ = 280 nm for indole compounds, 238 nm for lovastatin, and 257 nm for ergothioneine. The concentration of oxybenzone was determined using RP-HPLC according to the procedure developed by Chawla et al. [29]. Briefly, the separation was carried out on Hitachi HPLC apparatus (Merck, Tokyo, Japan) equipped with an L-7100 pump and a Purospher^®^ RP-C_18_ (200 mm × 4 mm , 5 μm) column (Merck, Tokyo, Japan), using methanol–water (95:5, *v*/*v*) as a mobile phase, with a flow rate of 1 mL min^−1^ and UV detection at λ = 280 nm. The qualitative analysis of oxybenzone was performed by comparing the retention times of the peaks in the samples with the retention times of the standard. To confirm the presence of oxybenzone in the tested extracts, a standard solution was added to the samples. The presence of the tested compound in the sample was indicated by an increase in the peak height for the appropriate retention time. In addition, MS/MS analysis was also performed. The quantitative analysis of oxybenzone was carried out using the calibration curve method where the concentration of the standard substance was in the range from 0.025 to 0.25 mg mL^−1^. The analysis of phenolic compounds, L-phenylalanine, and sterols was performed using a Hitachi-Merck HPLC VWR liquid chromatograph from Darmstadt, Germany, as previously described [28]. The quantities of all analyzed compounds were reported in milligrams per 100 g of dry weight (d.w.).

### 2.7. UPLC Analysis

To the samples obtained after lyophilization of 100 mL of solution, 20 mL of methanol (for LC-MS LiChrosolv^®^; Supelco, St. Louis, MO, USA) was added and shaken for 30 min. Then, the methanol solutions were centrifuged (20 min, 4000 RPM). The supernatants were immediately analyzed. Analysis was carried out using the UPLC method (ACQUITY UPLC I Class System, Waters; column: ACQUITY UPLC BEH C18, 130Å, 1.7 µm, 2.1 mm × 50 mm; detector: QTof (Xevo G2-XS Waters)). Detailed data of the analytical procedure and the mobile-phase compositions have been described previously [30]. The details of the chromatographic separation were as follows. Briefly, the gradient elution program was set as follows: starting at 0.0 min with 90% solvent A, transitioning to 40% A at 6.0 min, then to 10% A at 8.0 min, maintaining 10% A until 8.5 min, and finally returning to 90% A at 9.0 min and holding until 10.0 min. Component A was H_2_O (for LC-MS Chromasolv^®^; Fluka, Buchs, Switzerland), with 0.01% HCOOH (98–100%, for LC-MS, LiChropur^®^; Sigma-Aldrich) and Component B was CH_3_CN (for LC-MS LiChrosolv^®^; Sigma-Aldrich) with 0.01% HCOOH. The flow rate was maintained at 0.300 mL per minute. Sample volumes of 0.5 µL and 1.0 µL were injected. The column temperature was set at 35 °C. Electrospray ionization in positive mode (ES+) was used as the ion source. The scan time was set to 0.1 s per scan. The mass spectrometer scanned from 50.0 Da to 600.0 Da. The maximum mass error allowed was 0.5 mDa. The instrument operated in both MS and MS/MS modes. Collision energy ranged from 0 to 25 eV. Leucine Enkephalin was used as the reference compound for single-point calibration. Data acquisition and analysis were performed using MassLynx v4.1 software. Organic products of oxybenzone biotransformation were identified by comparing chromatograms recorded on the QTof detector for samples after incubation and the control sample (incubated without oxybenzone). The protonated monoisotopic molecular masses [M + H]^+^ and their fragmentation spectra (with fragmentation energies ranging from 10 to 25 eV) were determined for peaks corresponding to oxybenzone transformation products. The structural formulas of identified compounds were determined using the ChemDraw Std (ver. 23.1.1) with Analysis package (CambridgeSoft). Low-molecular-weight aliphatic reaction products were not identified.

### 2.8. Statistical Analysis

Statistical analyses were conducted using Microsoft Excel 2010 and GraphPad Prism v3.02 (GraphPad Software, San Diego, CA, USA). Three replicates were used for each sample. The results are expressed as means and standard deviation (SDs). One-way analysis of variance test with Tukey’s post hoc test of multiple comparisons was applied. Values were considered significantly different at *p*  <  0.05. Toxicity risk profiles of the biotransformation products were predicted in silico using OSIRIS Property Explorer (access: https://www.organic-chemistry.org/prog/peo/, accessed on 1 January 2020).

## 3. Results

*Lentinula edodes* (commonly known as shiitake) is extensively cultivated worldwide, including in Europe, due to its high culinary and medicinal value. *L. edodes* is effective in bioremediation due to its high enzymatic activity. It degrades lignin, eliminating pollutants like polycyclic aromatic hydrocarbons, dyes, pesticides, and phenolic compounds. Its oxidoreductase enzymes, such as laccases and manganese-dependent peroxidases, are crucial for degrading organic pollutants. *L. edodes* was selected for the study due to its proven efficacy in bioremediation of xenobiotics, including antifungal agents such as clotrimazole, bifonazole, and terbinafine [24,25]. At the outset of the study, the focus was on the analysis of the content of organic compounds in the fungal material. Subsequently, the content of identified antioxidant compounds was compared between the control and in vitro cultures of *L. edodes* treated with oxybenzone. The metabolic processes of oxybenzone within L. edodes were thoroughly investigated, and the impact of this compound on mycelium growth was included to understand the possible pathways of oxybenzone biotransformation in natural environments and its effects on living organisms.

### 3.1. Antioxidants Profile

The organic compound content in the *L. edodes* mycelium from in vitro cultures post-cultivation was examined to determine if oxybenzone could impact the antioxidant profile by altering the production of bioactive compounds (Table 1). The dry matter results for *L. edodes*, both with and without the addition of BP-3, showed statistically significant differences in the levels of L-tryptophan, p-hydroxybenzoic acid, ergosterol, lovastatin, and ergothioneine. L-tryptophan and ergothioneine were found in small amounts in the control mycelium, and their levels increased in the presence of oxybenzone after cultivation. Lovastatin was no longer present in the mycelium after cultivation with oxybenzone. Phenylalanine and the indole compound 6-metylo-D,L-tryptofan ware found in the same amounts in both the control mycelium and those with the addition of oxybenzone. Ergosterol and the phenolic compound p-hydroxybenzoic acid were found in the highest amounts in the control mycelium. Among the investigated organic compounds, phenylalanine was dominant. The investigation into the organic compound content indicated that BP-3 had a varied effect on the levels of organic compounds in *L. edodes* material. The observed increase in the content of some antioxidants, while reducing the content of others, such as lovastatin, suggests that the impact of xenobiotics on the antioxidant profile of in vitro cultures of *L. edodes* is complex.

### 3.2. The Impact of Oxybenzone on Mycelium Growth

**Figure 1:**
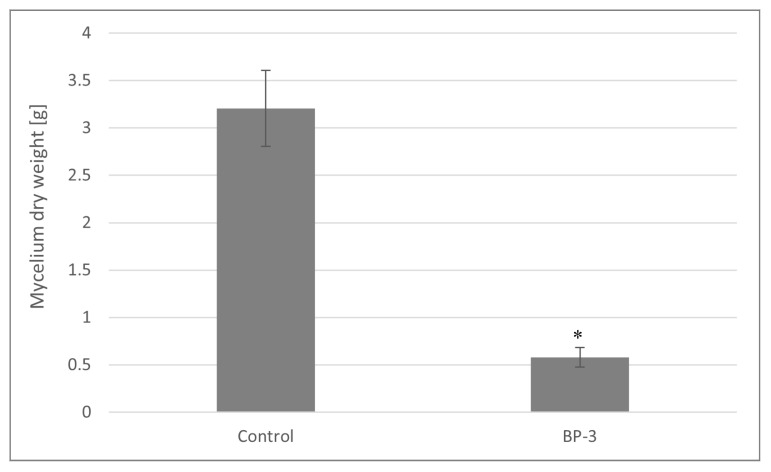
Comparison of the dry matter content of *L. edodes* mycelium from in vitro cultures supplemented with oxybenzone powder at a final concentration of 0.1 mg/mL. Statistical analyses were performed using one-way ANOVA followed by Tukey’s test. A significant difference from the control group (which did not receive oxybenzone) was marked with (*), *p* < 0.05, *n* = 3.

This study involved comparing the dry matter content of *L. edodes* mycelium from in vitro cultures, which were enhanced by the addition of oxybenzone powder. Figure 1 illustrates a comparison of the final average dry biomass obtained from in vitro cultures of *L. edodes* enriched with oxybenzone at the final concentration of 0.1 mg/mL (25 mg of oxybenzone powder) and without the addition of oxybenzone (control). The mycelium’s dry weight exhibited statistically significant differences between the control group and the fungal sample treated with oxybenzone powder (from 3.205 g ± 0.4022 to 0.5803 ± 0.1019 g for *L. edodes*) (*p* < 0.05). The addition of oxybenzone caused a significant inhibition of *L. edodes* mycelium growth. The addition of oxybenzone in powder form, which has poor solubility in water, likely provided prolonged availability of the compound for the remediation process. Consequently, the presence of oxybenzone might negatively impact the growth and development of the biomass by disrupting normal cellular functions. In previous studies conducted by Kryczyk et al., no effect of the antifungal agents clotrimazole and bifonazole, added in analogous amounts into in vitro cultures of *L. edodes*, on dry biomass weight was demonstrated [2019], and a small, but statistically significant, effect was noted for terbinafine powder [2020].

### 3.3. Mycoremediation of Oxybenzone

**Figure 2:**
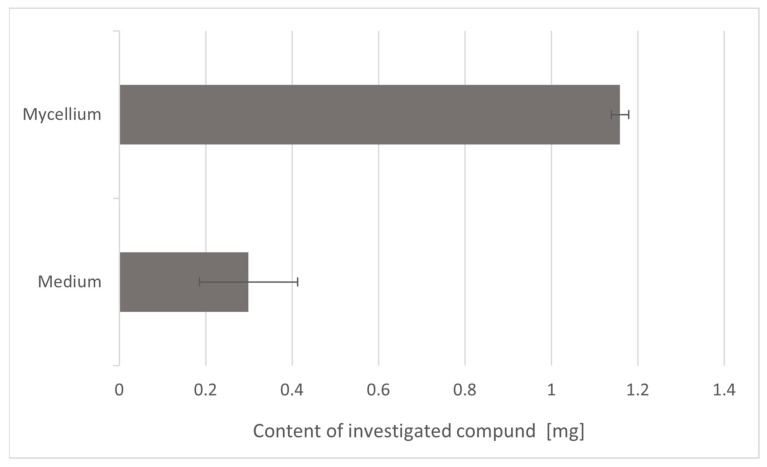
The total content of oxybenzone in the dry matter of mycelium and in Oddoux liquid medium obtained from in vitro cultures of *L. edodes* enriched with oxybenzone (25 mg) after 2 weeks of cultivation.

The primary objective of this research was to evaluate the efficacy of *L. edodes* in the biodegradation of oxybenzone. The results obtained in the study confirm the viability of Lentinula species for the degradation of oxybenzone. Figure 2 shows a comparison of the final average milligrams of BP-3 absorbed by mycelium after 2 weeks of growth and the final amount of oxybenzone determined in Oddoux liquid medium. It is clear that *L. edodes* significantly metabolized oxybenzone (BP-3). The initial amount of BP-3 in the Oddoux liquid medium was notably reduced (*p* < 0.05, n = 3). A considerable reduction in oxybenzone amounts was observed in the medium after incubation, decreasing from 25 mg to 0.2993 ± 0.1934 mg. After lyophilization, the final amount of oxybenzone in the mycelium was 1.1591 ± 0.0323 mg. The advantages of mycoremediation using in vitro cultures of *L. edodes* include its low cost and effectiveness. The degradation time could be reduced through increased density of the fungus. It can be concluded that *L. edodes* in vitro cultures were effective in degrading BP-3; however, they exhibited significantly lower growth compared to the control. *L. edodes* was chosen for study due to its proven efficacy in the biotransformation of antifungal agents [24,25]. In the case of terbinafine and azole antifungals, an experiment conducted under similar conditions showed significantly lower efficiency of the biotransformation process. The estimated total terbinafine content in the dry weight of mycelium post-cultivation was 7.63 ± 0.45 mg for the powder samples and 12.52 ± 2.46 mg for the cream samples [24]. The average total amount of bifonazole accumulated in the mycelium from *L. edodes* in vitro cultures was 9.22 mg for the bifonazole powder and 3.82 mg for the bifonazole cream [25].

### 3.4. Identification of Biodegradation Products of Oxybenzone and Toxicity Evaluation

**Figure 3:**
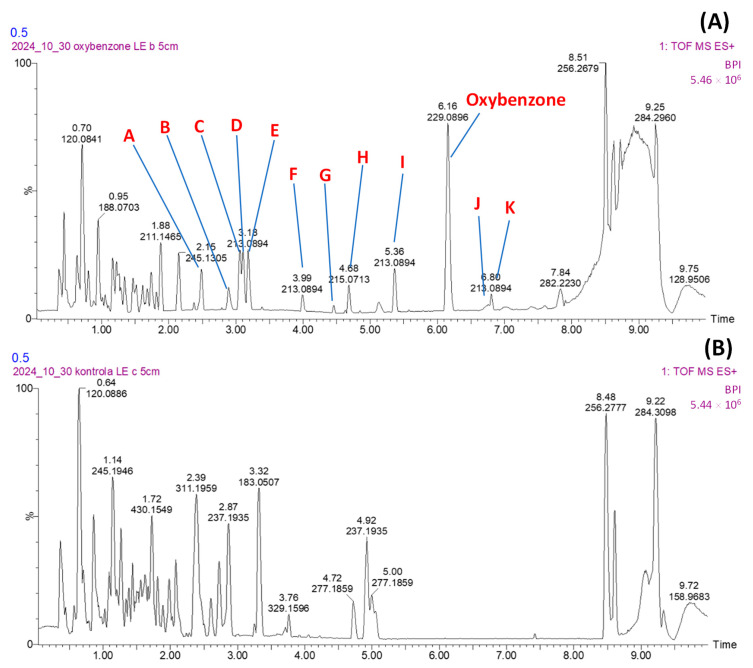
A representative chromatogram of the analyzed extracts of mycelium from in vitro cultures of *L. edodes* grown with (**A**) and without (**B**) oxybenzone. The identified products of oxybenzone biotransformation were described as A–K.The degradation mechanisms of oxybenzone described so far include hydrolysis, photoinitiated degradation, and biodegradation. The substance has been shown to undergo hydrolysis in laboratory experiments following the OECD 111 test guideline, but at a relatively slow rate. The rate of hydrolysis is dependent on pH, with the hydrolysis half-life at 25 °C determined to be approximately 82.4 days at pH 4, 41.9 days at pH 7, and 407 days at pH 9. Hydrolysis involves the chemical breakdown of a compound due to reaction with water, and this process can vary significantly with changes in pH levels [8]. Previous studies by Vione et al. identified benzoic acid and benzaldehyde as the primary photoproducts of BP-3 photolysis in bulk solution. These compounds result from the cleavage of the carbon–carbon bond that connects the carbonyl group to the aromatic ring containing methoxy and hydroxyl groups. 2,4-dihydroxybenzophenone (BP-1), formed by O-demethylation, was also identified as a biodegradation product A [31]. Cooper et al. described that the main photodegradation products are benzophenone, 2-hydroxybenzophenone, and 4-methoxybenzophenone. The last one was identify also as a mycoremediation product (product C), which was formed by removing the hydroxyl group from oxybenzone [32]. It is important to note that benzophenone, a known carcinogen, was not detected in mycelium and medium. Chen et al. identified two metabolites of BP-3 resulting from phytotreatment (uptake and metabolism of oxybenzone by hairy root culture of *Armoracia rusticana*): oxybenzone-glucoside and oxybenzone-(6-O-malonyl)-glucoside [33]. Furthermore, benzophenone-1 (BP-1) was produced from BP-3 as a biodegradation product in an oxygen environment [5]. There are studies evaluating the impact of benzophenone-type UV filters on the photodegradation of co-existing compounds in water. Specifically, Kodikara et al. have shown that the presence of benzophenone and its derivative, oxybenzone, significantly enhances the degradation rate of sulfamethoxazole by promoting the production of reactive intermediates [34].

**Figure 4:**
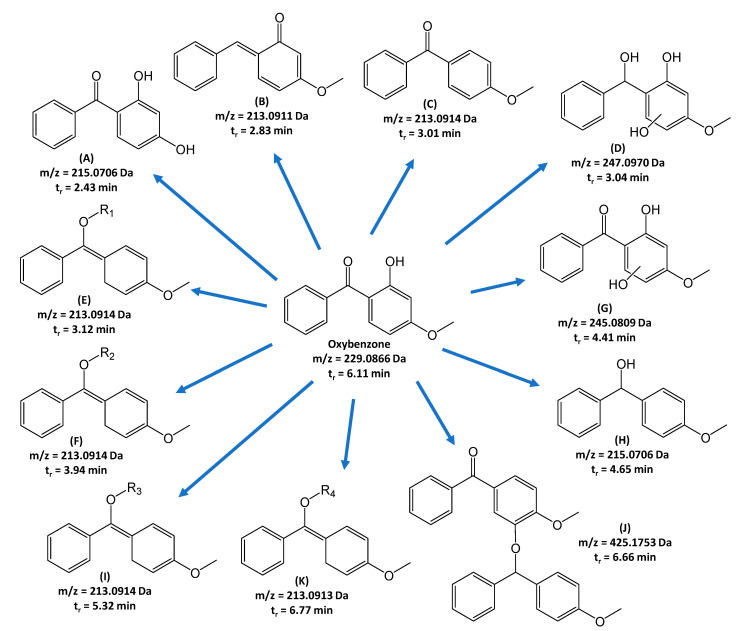
Identified biodegradation products formed in the mycelium and the medium obtained from in vitro cultures of *L. edodes* grown with the addition of oxybenzone.

In the study, extracts of mycelium from in vitro cultures of *L. edodes* were analyzed to assess the impact of oxybenzone addition. The cultures were grown both with and without the addition of oxybenzone. The comparative analysis highlights the variations in the mycelial composition under different growth conditions. The products of oxybenzone biotransformation were identified by comparing QToF detector chromatograms from incubated samples with (Figure 3A) and without (Figure 3B) oxybenzone, as described previously for sulfonamides [30]. The identified products formed after incubation of oxybenzone with in vitro cultures of *L. edodes* are shown in Figure 4 and Table 2. In the processes initiated by *L. edodes*, hydroxyl derivatives of oxybenzone and esterification products are formed—attaching large organic groups to the oxygen of the reduced carbonyl group. Condensation of two oxybenzone molecules was also possible (product J).

In addition, it is worth paying attention to the different concentrations of oxybenzone determined in the environment. Oxybenzone concentrations in water bodies can range from 0.01 to 1.4 µg/L. Higher concentrations are often found near areas with heavy recreational use or wastewater discharge. Furthermore, local practices, such as the use of personal care products containing benzophenone, can influence its concentration in the environment. Discrepancies could arise from differences in seasonal variations, geographical locations, and sampling extraction methods. In soil, oxybenzone concentrations have been reported to range from 0.1 to 2.5 mg/kg. These values can vary based on factors such as proximity to urban areas and agricultural practices [35,36].

Oxybenzone has been associated with several harmful effects in humans. It has been indicated that oxybenzone can act as an endocrine disruptor, potentially affecting hormonal balance. Additionally, it has been shown to be absorbed in significant amounts through the skin and passes into breast milk [37]. The potential for increased adverse reactions is also related to the contamination of fish with oxybenzone and/or its presence in drinking water [38]. Moreover, oxybenzone poses a hazard to coral reefs as it acts as a phototoxicant and genotoxicant to corals. Its levels in coral reefs in the U.S. Virgin Islands were found to be between 75 µg/L and 1.4 mg/L, while Hawaiian sites had concentrations ranging from 0.8 to 19.2 µg/L [36]. Therefore, we evaluated the predicted toxicity risks of oxybenzone and its degradation products using the OSIRIS Property Explorer. The assessment focused on their potential to induce mutagenicity, tumorigenicity, irritation, and reproductive effects (Table 3). Results showed that biodegradation products have a lower theoretical mutagenic, tumorigenic, and reproductive effect than oxybenzone. Due to the unknown structure of substituents R1-R4, the toxicity risks of products E, F, I, and K were not assessed. However, the fragment present in their structure derived from oxybenzone was evaluated.

## 4. Conclusions

Oxybenzone has been controversial due to its potentially negative impact on human health and the environment. In this paper, the impact of this UV filter on the antioxidant profile and biomass growth in in vitro cultures of *L. edodes* was demonstrated. Cumulatively, the findings suggest that the removal processes of oxybenzone, including uptake and biodegradation, were highly efficient. However, *L. edodes* in vitro cultures showed reduced growth, but the final concentration in the medium, as well as in the dry mycelium, was very low. Additionally, eleven products of oxybenzone biotransformation were identified. Mycoremediation is cost-effective, and the degradation time could be reduced by increasing the density of the fungus. To apply this method in real-world conditions, further efficiency-related tests would be necessary. Further research on the long-term effects of oxybenzone exposure on *L. edodes*, as well as the investigation of synergistic effects between oxybenzone and other cosmetic contaminants on fungal metabolism and growth, is required to provide a more comprehensive understanding of the environmental fate of organic UV filters.

## Figures and Tables

**Table 1:** Content of organic compounds in the *L. edodes* mycelium from in vitro cultures post-cultivation with the addition of BP-3 and without the addition of a UV filter [mg/100 g d.w.].

Mushroom Material		*Lentinula edodes* Mycelium from In Vitro Cultures	*Lentinula edodes* Mycelium from In Vitro Cultures with Oxybenzone
	Analyzed Compounds		
**Indole Compounds**			
L-Tryptophan		0.174± 0.023	17.08 ± 0.17 *
6-metylo-D,L-tryptofan		0.97 ± 0.02	1.20 ± 0.006
**Phenolic Compounds**			
p-Hydroxybenzoic acid		1.75 ± 0.09	0.98 ± 0.036 *
**Sterols**			
Ergosterol		147.3 ± 4.8	66.15 ± 0.54 *
**Other Organic Compounds**			
Lovastatin		22.06 ± 0.48	nd *
Ergothioneine		19.62 ± 0.85	41.10 ± 2.3 *
Phenylalanine		177.25 ± 5.11	163.47 ± 7.26

nd—not detected. Values in a row followed by * are different at *p* ≤ 0.05 (statistical analysis: one-way analysis of variance (ANOVA), followed by Tukey’s test). Each value represents the mean of three replicates ± standard deviation.

**Table 2:** Detailed data of LC-MS/MS analysis of solutions after *L. edodes* cultivation.

Retention Time (t_r_) (min)	Symbol in Figure 3 and Figure 4	Parent Ions [m/z]	Summary Formula [M + H^+^]	Daughter Ions [*m*/*z*]; Formula
2.43	A	215.0706	C_13_H_11_O_3_	137.0235; C_7_H_5_O_3_ 105.0345; C_7_H_5_O
2.83	B	213.0911	C_14_H_13_O_2_	198.0677; C_13_H_10_O_2_ 181.0652; C_13_H_9_O 170.0730; C_12_H_10_O 153.0702; C_12_H_9_ 152.0626; C_12_H_8_
3.01	C	213.0914	C_14_H_13_O_2_	198.0677; C_13_H_10_O_2_ 181.0652; C_13_H_9_O 170.0730; C_12_H_10_O 153.0702; C_12_H_9_ 152.0626; C_12_H_8_
3.04	D	247.0970	C_14_H_15_O_4_	229.0858; C_14_H_13_O_3_ 217.0857; C_13_H_13_O_3_ 151.0400; C_8_H_7_O_3_ 105.0345; C_7_H_5_O
3.18	E	213.0914	C_14_H_13_O_2_	198.0677; C_13_H_10_O_2_ 181.0652; C_13_H_9_O 170.0730; C_12_H_10_O 153.0702; C_12_H_9_ 152.0626; C_12_H_8_
3.99	F	213.0914	C_14_H_13_O_2_	198.0677; C_13_H_10_O_2_ 181.0652; C_13_H_9_O 170.0730; C_12_H_10_O 153.0702; C_12_H_9_ 152.0626; C_12_H_8_
4.41	G	245.0809	C_14_H_13_O_4_	167.0357; C_8_ H_7_ O_4_ 151.0400; C_8_ H_7_ O_3_ 105.0345; C_7_ H_5_ O
4.68	H	215.0706	C_13_H_11_O_3_	137.0235; C_7_H_5_O_3_ 105.0345; C_7_H_5_O
5.36	I	213.0914	C_14_H_13_O_2_	198.0677; C_13_H_10_O_2_ 181.0652; C_13_H_9_O 170.0730; C_12_H_10_O 153.0702; C_12_H_9_ 152.0626; C_12_H_8_
6.16	Oxybenzone	229.0866	C_14_H_13_O_3_	151.0400; C_8_H_7_O_3_ 105.0345; C_7_H_5_O
6.68	J	425.1753	C_28_H_25_O_4_	393.1479; C_27_H_21_O_3_ 213.0913; C_14_H_13_O_2_ 91.0552; C_7_H_7_
6.80	K	213.0913	C_14_H_13_O_2_	198.0677; C_13_H_10_O_2_ 181.0652; C_13_H_9_O 170.0730; C_12_H_10_O 153.0702; C_12_H_9_ 152.0626; C_12_H_8_

**Table 3:** The toxicity risk assessment of oxybenzone and its biotransformation products evaluated in *L. edodes* mycelium cultured in media enriched with oxybenzone. Predictions were made using OSIRIS Property Explorer.

Compound	Mutagenic	Tumorigenic	Irritant	Reproductive Effects
**Oxybenzone**	known to be mutagenic	known to be tumorigenic	–	high-risk fragment
**Product A**	high-risk fragment	medium-risk fragment	high-risk fragment	medium-risk fragment
**Product B**	–	–	–	–
**Product C**	–	–	–	–
**Product D**	–	–	–	–
**Product G**	–	–	high-risk fragment	–
**Product H**	–	–	–	–
**Product J**	–	–	–	–

## Data Availability

The original contributions presented in this study are included in the article. Further inquiries can be directed to the corresponding author.
